# Physicians Educate Your Patients

**Published:** 1859-10

**Authors:** O. C. Tooker

**Affiliations:** Greensburg, Ohio


*Physicians educate your Patients.*—There is nothing more vexatious to the practitioner of medicine than the profound ignorance with which he meets in his community, as it exists among his patients to whom he is daily prescribing. I mean their ignorance of medicine and its effects upon the human system. A physician is called to visit and prescribe for a patient, laboring under a bilious remittent fever, for instance; he calls, examines the pulse, looks at the tongue, and makes up his diagnosis from the symptoms manifested, and from whatsoever he can learn from inquiries made; he prescribes, deals out his medicine, and perhaps says he will call the next day. He leaves the patient, and the attending nurse, without telling them what effect he expects to get from the medicine given, whether he expects them to sweat freely, or whether their bowels are to move or not, or how the feces should look under the operation of the physic, and much less does he inform them of the kind and character of the medicine he leaves them, for many would rather attach long and almost unpronounceable names, as if there was a magic in the name that would work a miracle in their case; and therefore the patient is left wholly in the dark; the physician has been home but a short time, before a servant is sent for the doctor again in a great hurry, for the patient is no better, but has a very severe pain in his bowels, his fever is higher and his head pains him worse, and the patient himself is expecting to die almost momentarily. The doctor makes haste and goes in the house only to find that his severe pain is only in consequence of the action of the physic, the bowels have not moved, and the fever is about the same as before. He soon quiets the patient by telling him, that his symptoms are no worse, that it is no less than might have been expected from the action of the medicine given him, for his stomach was very much out of order, &c., &c. The patient replies, Well, I did not know what you gave me to take, nor whether the medicine would have this effect upon me or not, nor what the matter was; but I thought I would send and have you come up. If you had told me this before, I

wouldn't have been alarmed, it would have saved all this trouble. Again, the doctor may administer a dose of calomel to his patient with no particular directions; and the patient takes cold, and through his imprudence he gets a sore mouth ; and the doctor is always and invariably to blame, not so much for giving the calomel, but for not telling the patient of it and giving proper precautions.

I believe there is no greater obstacle to the success of the physician than this ignorance, caused by a failure upon his part to instruct the patient and the family in regard to these things, which are of so much importance. If the physician would make it a practice of instructing people and communities in relation to this, he would avoid a great many *unnecessary* calls, and thereby escape the complaints of large bills, &c., &c., beside being much more successful in the cure of disease. Teach the people, when practicable, the properties of your medicines, and how to use them, and under what circumstances, &c., &c.; and they will not only assist very materially in the cure of disease, but you will soon have an enlightened community, in which it is always pleasanter to practice medicine, and do away with that superstition and ignorance upon which quacks feed and depend for their support. Yes, if you would do away with *quackery* and superstition, educate the people, enlighten the communities, and instruct in these things, and you will pave the way for the more rapid advancement of the science of medicine.

Greensburg, Ohio. Yours truly,

O. C. Tooker, M. D.

